# Ethical issues of unrelated hematopoietic stem cell transplantation in adult thalassemia patients

**DOI:** 10.1186/1472-6939-12-4

**Published:** 2011-03-08

**Authors:** Giovanni Caocci, Giorgio La Nasa, Ernesto d'Aloja, Adriana Vacca, Eugenia Piras, Michela Pintor, Roberto Demontis, Salvatore Pisu

**Affiliations:** 1 Department of Hematology, University of Cagliari, Cagliari, Italy; 2 Department of Forensic Medicine, University of Cagliari, Cagliari, Italy; 3 Mediterranean Clinical Center of Bioethics, University of Cagliari-Sardegna Ricerche, Italy

## Abstract

**Background:**

Beta thalassemia major is a severe inherited form of hemolytic anemia that results from ineffective erythropoiesis. Allogenic hematopoietic stem cell transplantation (HSCT) remains the only potentially curative therapy. Unfortunately, the subgroup of adult thalassemia patients with hepatomegaly, portal fibrosis and a history of irregular iron chelation have an elevated risk for transplantation-related mortality that is currently estimated to be about 29 percent.

**Discussion:**

Thalassemia patients may be faced with a difficult choice: they can either continue conventional transfusion and iron chelation therapy or accept the high mortality risk of HSCT in the hope of obtaining complete recovery.

Throughout the decision making process, every effort should be made to sustain and enhance autonomous choice. The concept of conscious consent becomes particularly important. The patient must be made fully aware of the favourable and adverse outcomes of HSCT. Although it is the physician's duty to illustrate the possibility of completely restoring health, considerable emphasis should be put on the adverse effects of the procedure. The physician also needs to decide whether the patient is eligible for HSCT according to the "rule of descending order". The patient must be given full details on self-care and fundamental lifestyle changes and be fully aware that he/she will be partly responsible for the outcome.

**Summary:**

Only if all the aforesaid conditions are satisfied can it be considered reasonable to propose unrelated HSCT as a potential cure for high risk thalassemia patients.

## Background

Beta thalassemia major is a severe hereditary hemolytic anemia that arises from reduced or absent synthesis of the hemoglobin subunit beta. Clinical presentation occurs between the ages of 6 months and 2 years. Red cells are rapidly destroyed, freeing large amounts of iron that are deposited in organs and tissues. Hyperabsorption of iron by the gastrointestinal tract, driven by the ineffective erythropoiesis, contributes to hemochromatosis-induced organ damage. Iron overload is furthermore aggravated by the frequent transfusions required to maintain blood-oxygen carrying capacity in these patients. Sequelae include severe anemia, hepatic fibrosis and cirrhosis, diabetes mellitus, hypogonadism, growth retardation, sexual immaturity, moderate to severe pulmonary syndromes and cardiac disorders. Myocardial disease is by far the most important life-limiting complication and is responsible for about 70 percent of deaths in these patients [1].

The survival of patients with thalassemia major is continuously improving, but despite the advances made in iron chelation therapy [2,3], the prevalence of severe complications such as heart failure, arrhythmias, and diabetes remains high. Allogenic HSCT remains the only potentially curative treatment for patients with thalassemia [4]. The outcome of HSCT partially depends upon the patient's pre-transplantation clinical conditions, particularly the extent of hepatomegaly and/or liver fibrosis and the magnitude of iron accumulation. An HLA identical sibling is the donor of choice for patients requiring allogenic HSCT. After successful HSCT, patients receive regular phlebotomy therapy until iron stores return to normal [5]. When an HLA-matched sibling donor is not available, HSCT from an unrelated voluntary donor is a feasible alternative provided that the donor is selected according to stringent HLA compatibility criteria. Experience has shown that by applying highly stringent criteria for donor selection, the outcome of HSCT is comparable to that obtained when the donor is a compatible sibling [6]. Unfortunately, adult thalassemia patients belonging to risk class 3 of the Pesaro classification (patients with hepatomegaly, portal fibrosis and a history of irregular iron chelation) have an elevated risk for transplantation-related mortality (TRM) which is currently estimated to be about 29 percent [7]. The experience acquired in our bone marrow transplantation center confirms this percentage. So far, 36 high risk thalassemia patients (16 males and 20 females) aged 16 years or older (median 22 yrs, range 16 - 37 yrs) have received HSCT in our Center. Five-year overall survival (median follow-up period of 5.8 years) was 75% while the cumulative incidence of TRM was 25%. Nine patients died of transplantation-related causes. Furthermore, 21.5% of the survivors developed chronic graft-versus-host disease (GvHD).

## Discussion

A decisive choice

All things considered, the clinical hematologist is faced with a multitude of risk factors (disease severity, early onset, the difficult management and treatment of complications of beta- thalassemia major) as well as the demanding task of identifying patients likely to incur a bad outcome [8]. Unfortunately, it is difficult, if not impossible, to foresee the outcome for each patient. Indeed, our ability to predict the burdens and benefits of HSCT is very poor. A well known method is to stratify patients from risk class 1 (5% of mortality) to risk class 3 (29% of mortality) of the Pesaro classification. After that, all the elements we possess to communicate risk to the patient are statistic. Unfortunately, a 29% mortality risk could be perceived by the patient as a severe threat. Therefore, the doctor's ability to communicate the risks and benefits and the patient's capacity to understand both the elements of risk and the significance of the transplantation procedure are crucial to the ultimate decision of what the patient must do.

Hence, the patient is faced with a decisive choice: he/she can either undergo HSCT with a good probability of **cure **but a high chance of death or continue to rely on conventional transfusion and chelation therapy. From this it follows that the choice of HSCT needs to be sustained by in-depth knowledge of the quality of life of both transplanted and untransplanted patients. The WHO defines quality of life (QoL) as: « an individual's perception of their position in life in the context of the culture and value systems in which they live and in relation to their goals, expectations, standards and concerns. It is a broad ranging concept affected in a complex way by the person's physical health, psychological state, personal beliefs, social relationships and their relationship to salient features of their environment» [9]. Some of the major clinical and psychological aspects of thalassemia are: the effect of chronic illness on family stability and dynamic family structure, bone deformities and short stature leading to poor self image, frequent hospital appointments for transfusions, overnight subcutaneous infusions, poor sexual development, impaired fertility, disease or therapy-related complications and uncertainties about the future [10]. Additional psychological and social problems may arise from interference of the aforesaid aspects with employment and education. Overall, despite the progress made, prolonged dependency on family care continues to jeopardize these patients' right to self determination and autonomy.

None the less, patients who are compliant with conventional treatment modalities can expect to enjoy a good quality of life with a life expectancy of about 50 years. The life expectancy in the era of new chelators, other therapies and non-invasive diagnostics is not yet known. A recent paper published by Caterina Borgna-Pignatti [11] provides valuable data on the quality of life of patients with thalassemia major and the survival rates of the different birth cohorts.

On the other hand, HSCT offers a definitive cure but is burdened by significant morbidity and mortality. Even if HSCT is successful, the risk of serious complications such as acute and chronic GvHD remains. Chronic GvHD can have a negative impact on an individual's general health and mental health, and can lead to the development of functional impairment and activity limitations. We also underscore that the QoL and overall health of individuals who have been successfully treated for chronic GvHD are not different from those with no history of chronic GvHD. Additionally, endocrinopathies, particularly gonadal dysfunction, can still occur. Therefore, it becomes crucial to consider the dilemmas that arise from the choice of HSCT in order to emphasize under what conditions this therapeutic approach may be considered the best choice for the patient.

### Ethical issues concerning the principles of beneficence, non maleficence and autonomy

Regular blood transfusions and iron chelation therapy have transformed thalassemia from a rapidly fatal disease to a chronic disorder compatible with prolonged survival. The recent availability of oral iron chelators and the progress made over the past 30 years toward understanding the genetic defects and pathogenic mechanism underlying thalassemia syndromes, has led to the development of innovative therapeutic procedures that have substantially improved the life expectancy and the quality of life of affected patients [12,13]. However, despite the advances made, complications such as inadequate transfusions, transfusion transmitted viral diseases, allo-sensitization, liver and cardiac disturbances and iron chelation toxicity, keep arising. Moreover, additional previously undescribed complications (thromboembolic episodes and atherogenesis-related pathologies) are now being recognized. Alternatively, the elective option of HSCT can greatly enhance quality of life, if not only by removing the inconvenience caused by iron chelation and transfusion therapy. Within this context, several ethical dilemmas become apparent:

a - Is it reasonable to consider HSCT a feasible option, capable of achieving the good of the patient in a situation where the probability of harm is very high?

b - How can we put the patient in a condition to choose freely? In other words: how can physicians fully respect and support the patient's autonomous choice.

c - Is every patient a good candidate for HSCT or is it mandatory to screen for suitable patients and, if so, which criteria should be used?

### The good of the patient

It is the undeniable duty of the physician to do what is best for the patient. The concept of good care is firmly rooted and represents a widespread aim in medical science. Although unrelated HSCT makes it possible to achieve this goal, the risk of maleficence is high. Therefore, even after the informed consent process has been completed and the therapeutic procedure has started, patient-physician communication must continue in order to reach the best outcome. The ethical concept founding the physician's task may be well expressed by the golden rule: *treat others only in ways that you're willing to be treated in the same exact situation *[14]. As Sydenam said, we offer to the sick man the same therapeutic options as we would claim for ourselves. Our experience in the clinical setting suggests that a fair decision cannot exclusively be based on the physician's clinical judgement. In taking the decision, the physician must come to terms with the needs of the patient. The patient is a unique and valuable person and not just a disease to be treated. Albeit scientific knowledge of the disease is essential, it is only an element of clinical judgement. A high standard of patient care can only be achieved if the physician applies a more holistic approach. In fact, the healing relationship is not simply a contract based on scientific authority, but presupposes the virtue of the physician to acknowledge the "attributes of the patient as a person" [15]. The issue is not simply "to cure" but "to take care" of the patient [16].

Alongside the clinical findings, it is of fundamental importance to build a physician-patient relationship based on the virtues of prudence, sharing, attention, willingness and so on. Above all, it is mandatory to individuate the main moral problem requiring attention. As mentioned previously, there is a high probability of maleficence for patients undergoing HSCT: initially, the large majority suffer relevant worsening of their already endangered health and about 25 percent die within one year [17]. This does not make it easy to apply the Hippocratic rule "Primum non nocere", since there is an elevated risk (one every four patients) of severely harming - till his/her death - the patient. If we consider the central position of beneficence in medical ethics, HSCT would appear, at least prima facie, as being too hazardous to be suggested as a valid alternative to conventional treatment in high risk thalassemia patients. However, this is counterbalanced by the ability of HSCT to completely restore health, so much so that many patients consider themselves as "*reborn*" after HSCT and use the date of engraftment to celebrate their birthday [18]. In a pharmacological experimentation it would be unacceptable to tolerate such a high risk of unpleasant side effects in order to reach a beneficial outcome. And here we are not dealing with an advantageous result that may be burdened by the presence of some unpleasant side effects but we are faced with the probability of a potentially detrimental and irreversible consequence of treatment combined with absence of the desired outcome. Consequently, it can be said that unrelated HSCT for thalassemia is more similar to an "*all-or-nothing*" situation. In a life-threatening situation such as severe acute leukemia or lymphoma, where HSCT is used as a last resort to avoid death, it is beyond dispute that patients who have a suitable donor should be given the opportunity to undergo HSCT. However, the situation is quite different when the alternative is an expected lifespan of twenty years or more.

In the light of these considerations the problem is not only who is the best candidate to undergo to HSCT but, more radically, if the proposed procedure is consistent with the fundamental aim of medicine, that is beneficence. Any negative answer to this question would let the subject drop. For example, the eminent philosopher William Frankena treats the principle of beneficence as being divisible in four parts: a - One ought not inflict evil or harm; b - One ought to prevent evil or harm; c - One ought to remove evil or harm; d - One ought to do or promote the good [19]. These four sub principles are serially arranged so that in circumstances of conflict the first overrides the second, the second takes precedence over the third and so on. Being Frankena right, it would be hard to consider HSCT a good choice. Other Philosophers clearly distinguish between beneficence and non-maleficence. Obligations not to harm others are distinct from those to help others. Sometimes, the obligation not to harm others is more important than the one to promote the good, but in a changing context, the obligation to try to promote the good outweighs the concept of non-maleficence [20]. Given the fact that in many situations benefits cannot be achieved without inflicting harm, many writers in the field of medical ethics retain that one must accept substantial risk in order to safeguard life or restore health. If we consider the delicate balance between the harms and benefits of unrelated HSCT for thalassemia, it clearly emerges that the decision for HSCT must account for the fact that thalassemia patients have a valid alternative and that is, to continue their life with conventional therapeutic support. Yet, in the light of our previous observations, we retain that it is reasonable and appropriate for the physician to propose HSCT provided that the patient has a central role in the decision-making process in concordance with the moral principle of respect for autonomy.

### The autonomy of the patient

Decision-making in medicine requires the respect of individual freedom, dignity and moral values. In the past, the physician was the one to take the final decision, regardless of the relationship with the patient [21]. Physicians had a paternalistic approach, recommending what they felt was best for the patient. The growth of liberalism with its many social changes has been a challenge to paternalism over the last few decades. The new paradigm is founded on the respect for autonomy and high ranking of principles and today represents one of the most important guides to reasoning in bioethics. Autonomous action is analyzed in terms of normal choosers who act: 1 - intentionally; 2 - with understanding; 3 - without controlling influences that determine their action.

Physicians are more and more aware that they cannot heal a person solely by curing the disease. Beneficence cannot be achieved without full respect for patient autonomy. The patient should always be given the last word and be entitled to freely accept or decline the proposed therapeutic measures and/or treatment options. No-one should be coerced into unwanted behaviours. We completely agree with the statement made by George Surbled in 1895: «the patient has the right to refuse any kind of trial or therapy simply barricading himself/herself behind his/her wish, without presenting any justification» [22]. Hence, the responsibility of the physician is to provide all the elements that will assist the patient in the decision-making process. A well-pondered choice and conscious consent go hand in hand. Consent is essential to patient autonomy, not only intended as a capacity for independent decision and action but, above all, conscious decision and action [23]. The pursuit of conscious consent is paramount and the physician needs to be reminded that respecting the freedom of the patient is part of the goal of his profession and that awareness is the only ground where freedom can be respected.

Consciousness encompasses free will and independence of choice. We agree with Paul Ramsey when he portrays consent as "the cardinal canon of loyalty joining men together in medical practice and investigation" [24]. In the document *Information and Consent for Anesthesia*, issued by the Association of Anesthetists of Great Britain and Ireland, the term "informed consent" is replaced with "express consent" [25]. Although the authors clearly distinguish between the duty to obtain informed consent and the obligation to warn patients of the risks of a procedure, a considerable amount of ambiguity remains. The consent may be informed, clear, precise and explicit, and yet fail to fulfill the most crucial task: patient awareness. Shared decision-making is of fundamental importance when considering the option of HSCT. The physician's role is to provide a clear picture of the patient's current clinical situation and to carefully explain what the situation is likely to be in the future. The physician should aim at developing a high level of mutual understanding with the patient. Only if the patient is made fully aware of the possible risks and benefits of a proposed treatment is it possible to respect autonomy and consciousness. In the case of HSCT the patient's autonomy may be undermined in several ways. Bias in the perception of risks can lead the patient to misunderstand the advantages and disadvantages of HSCT. The understandable desire for recovery may induce the patient to perceive only the positive aspects and to neglect the real risk of danger of the procedure; at the same time the possibility of a definitive cure could lead the physician to emphasize the favourable outcomes of the procedure and to minimize the adverse effects. In both cases, the patient is unable to make a free and conscious choice. Furthermore, many people possess an unrealistic optimism that may lead them to believe that the specific risks are significantly less than objective probability might suggest [26]. There is plenty of evidence demonstrating that the information supplied during the decision-making process is often not well understood and, consequently, that the decision to undergo HSCT cannot be considered to be truly autonomous. Some studies suggest that people are more prone to understand qualitative rather than quantitative items [27]. Therefore, it should not be assumed that the supply of a large amount of information is the best way to ensure patient awareness. The difference between communicated and perceived data can be striking. This is not necessarily caused by poor communication but is rather due to the misunderstanding that the volume of information corresponds to its comprehension. Often when the physician supplies a large amount of information, the patient loses the central point; other times the amount of information may be appropriate but the physician is not sufficiently involved. Information can be useful in achieving awareness but it does not correlate with understanding. When the negative consequences of a proposed treatment strategy are discussed with the patient there is generally a rise in the discrepancy between the information conveyed by the physician and the information perceived by the patient [28]. This makes it important to emphasize the adverse and dramatic effects of HSCT, particularly the development of severe acute and/or chronic GvHD and the possibility of death. Communication skills also have their importance. A therapy reported to be 60 percent effective would be evaluated more favourably than a 40 percent failure rate, even though the two statements are objectively equal [29]. Preferably, the physician should use the negative statement to ensure that the patient fully grasps the meaning. This approach is more challenging but it is not by denying the risks that we act on the patient's behalf. The burden of chronic illness coupled to a desperate hope for recovery often leads the patient to underestimate the significance of adverse effects. The Medical Defense Union of London clearly asserts: "Doctors...have a duty to explain to the patient, in non-technical language, the nature, purpose and material risks of the proposed procedure" [30].

The following steps are essential to conscious consent: 1 - patients must be made aware of their overall medical condition; 2 - physicians must communicate the reasons for which the treatment is recommended; 3 - the benefits of HSCT must be clearly explained, but at the same time, therapy-related risks need to be vigorously stressed. The physician must scrupulously check that the patient has clearly and distinctly understood; 4 - finally, the physician needs to discuss the clinical features and prognosis of the patient in relation to conventional treatment schemes.

The process of understanding can be slow and laborious and makes it important for the physician to exert the virtues of prudence, patience, attentiveness and understanding to the best of his ability. Patients must be given sufficient time to take-in and ponder their choices. All these efforts combined should guarantee an acceptable level of awareness with full respect for patient autonomy. However, conscious consent only partially solves the problem. Another important ethical aspect is: Who is the best candidate for HSCT and who is responsible for enrollment? The second question is easy to answer since the physician is the only one to possess the necessary authority, knowledge and competence to decide who must or must not undergo HSCT. What calls for additional ethical reflection is the choice of the best candidate for HSCT. Whether a patient is suitable or not remains a difficult decision based on complex criteria, including objective and subjective data.

### The right patient

We believe that in order to choose the right patient it is not sufficient to consider only the clinical aspects. Such criteria would include too many patients and the information collected would be too general. We need to take into account other patient characteristics along with some environmental factors.

First of all, the physician must make sure that the patient possesses sufficient physical and psychological coping abilities to deal with all the complex aspects of unrelated HSCT.

The patient must be capable of understanding what the doctor is doing, why he/she is doing it, what the alternatives may be, and what the future will mean for the things he/she values most. The capability of understanding comprises at least three factors: natural sensibility, educational completeness and attentiveness. All these factors contribute to the assessment of the patient's competence. The concept of competence embraces the ability to understand information concerning important decisions related to one's health, to appreciate the significance of such decisions in relationship to one's own life as well as the ability to use the information to reason, choose and express one's choice [31].

The general rule could be expressed in this way: as much as the person is lacking in knowledge, motivation and capability of free decision, just as much should we be reluctant to enroll these patients for unrelated HSCT.

Furthermore, the physician needs to assess whether the patient has the capability to make the necessary lifestyle changes (for example, increased self care for adequate symptom management during the post-transplantation course). This is particularly true when patients develop chronic GvHD and rigorous therapy becomes mandatory. The patient is responsible for his/her health and investigation of the patient's attitude toward preserving health is an important part of the communication process. Wisdom in patients may be particularly crucial in contemporary medicine [32]. Another important aspect concerns the availability of competent support from significant family members or caregivers. The patient needs to receive loving care in a comfortable setting. Therefore, although the final decision lies with the patient, not every patient is suitable for HSCT. In order to choose the best candidates for HSCT, we must carefully consider all of the aforesaid elements. Synthetically, patients should have: 1 - extensive knowledge of the potential risks associated with the procedure; 2 -strong support from caregivers; 3 - a responsible attitude toward their health.

We believe that to setup a decision-making grid in an attempt to precisely "calculate" each of these elements is not the best way to decide. Rather, our opinion is that doctors ought to strive to be wise and, in particular, carefully assess all relevant elements in order to select the best patients.

In contemplating the use of healthy subjects in clinical trials, Hans Jonas, speaking about the process of informed consent, suggests the rule of "descending order". He affirms that the more patients lack in knowledge, motivations, freedom and responsibility, the more we must be prudent and thrifty [33]. In unrelated HSCT, this rule is particularly appropriate and could be said in this way: the more patients lack the three fundamental characteristics mentioned above, the more the physician needs to be careful in considering patients eligible for HSCT and, alternatively, should consider the possibility of continuing conventional therapy.

## Summary

The fundamental goal of medicine is to help a sick person in need and to enhance the quality of life of the individual being treated. HSCT is a procedure consistent with this goal and represents a valid alternative to the conventional treatment of thalassemia. However, considering that high risk thalassemia patients have an increased risk of treatment-related mortality, the concept of conscious consent becomes particularly important. Throughout the entire decision-making process, every effort should be made to sustain and enhance the autonomous choice of the patient. The physician must use communication skills that enable the patient to fully understand the favourable and adverse outcomes of the procedure. Although it is his duty to illustrate the possibility of completely restoring health, considerable emphasis must be put on the adverse effects of the procedure, particularly the possibility of dying and the development of severe acute and/or chronic GvHD. Finally, the physician must decide whether the patient is a suitable candidate for unrelated HSCT according to the "rule of descending order" that accounts for the patient's educational, social, cultural and family background besides his/her overall ability to comprehend. The combination of all these factors will ensure that patients fully understand the advantages and disadvantages of the procedure as well as the importance of competent support from relatives and/or caregivers during the pre- and post-transplantation period. The patient must be made aware that he/she will be partly responsible for the outcome. Full details on self-care and fundamental lifestyle changes are mandatory. Only if all these conditions are satisfied can it be considered reasonable to propose unrelated HSCT as a potential cure for high risk thalassemia patients.

## Competing interests

The authors declare that they have no competing interests.

## Authors' contributions

GC contributed to the clinical management of the patients, performed the statistical analysis and helped draft the manuscript; GLN contributed to the clinical management of the patients and participated in the design of the study; ED addressed the ethical and legal aspects and helped draft the manuscript; AV and EP contributed to the clinical management of the patients; MP addressed the legal aspects and helped draft the manuscript; RD addressed the legal aspects; SP contributed to the ethical issues and helped draft the manuscript. All authors read and approved the final manuscript.

## Pre-publication history

The pre-publication history for this paper can be accessed here:

http://www.biomedcentral.com/1472-6939/12/4/prepub
